# Prognostic factors in the first hour post admission for intra-hospital mortality in patients with septic shock in an ICU

**DOI:** 10.1186/cc12949

**Published:** 2013-11-05

**Authors:** Bárbara Magalhães Menezes, Fernanda Vilas Bôas Araújo, Fábio Ferreira Amorim, Adriell Ramalho Santana, Felipe Bozi Soares, Jacqueline Lima de Souza, Mariana Pinheiro Barbosa de Araújo, Louise Cristhine de Carvalho Santos, Pedro Henrique Gomes Rocha, Mateus Gonçalves Gomes, Osvaldo Gonçalves da Silva Neto, Pedro Nery Ferreira Júnior, Alethea Patrícia Pontes Amorim, Rodrigo Santos Biondi, Rubens Antônio Bento Ribeiro

**Affiliations:** 1 Escola Superior de Ciências da Saúde, Brasília, Brazil; 2 Liga Acadêmica de Medicina Intensiva de Brasília, Brazil; 3 Hospital Anchieta, Brasília, Brazil

## Background

Severe sepsis and septic shock are common and are associated with substantial mortality and substantial consumption of healthcare resources [1]. Although the incidence of septic shock has steadily increased during the past several decades, the associated mortality rates have remained constant or have decreased only slightly [2]. Our study aimed to identify the prognostic factors during the first hour after admission for intra-hospital mortality in patients with septic shock in a general ICU.

## Materials and methods

Case-control study conducted on patients admitted to the ICU of Hospital Anchieta, Brasília, DF, Brazil, during 17 months. Patients were divided into two groups during the hospital stay: survivors group (SG) and nonsurvivors group (NSG). Patients proceeding from or transferred to another ICUs were excluded.

## Results

During the period of the study, 1,918 patients were admitted, 120 with septic shock (6.2%). For this sample, the mean age was 62 ± 20, SAPS3 was 71 ± 18, 55.8% were males, and the hospital mortality was 49% (*n *= 59). In the NSG group, there was a higher incidence of decreased level of consciousness (83.1% vs. 52.5%, *P *= 0.00), acute respiratory failure (96.6% vs. 83.6%, *P *= 0.03), hypocapnia (60.8% vs. 39.2%, *P *= 0.02), acidemia (77.4% vs. 50%, *P *= 0.01), need for invasive mechanical ventilation (83.1% vs. 45.9%, *P *= 0.00), and thrombocytopenia <60,000/mm^3 ^(25.4% vs. 4.9%, *P *= 0.00). There was no difference between the groups regarding age (64 ± 21 vs. 61 ± 18, *P *= 0.37), PaO_2_/FiO_2 _ratio (174 ± 129 vs. 250 ± 236, *P *= 0.07), gender (47.5% vs. 63.9%, *P *= 0.37), incidence of acute renal injury (47.5% vs. 32.8%, *P *= 0.14), and arterial lactate (2.1 ± 1.1 vs. 1.8 ± 1.0, *P *= 0.13). The NSG group had greater SAPS3 score (82 ± 14 vs. 60 ± 15, *P *= 0.00). After logistic regression, only decreased level of consciousness (*P *= 0.00) and need for invasive mechanical ventilation (*P *= 0.01) were independently associated with hospital mortality.

## Conclusions

In patients with septic shock, decreased level of consciousness and need for invasive mechanical ventilation were independently associated with hospital mortality.
